# Technical standardization and total factor productivity in innovation-driven development: Evidence from China

**DOI:** 10.1371/journal.pone.0287109

**Published:** 2023-10-05

**Authors:** Shuo Wang, Yueping Zheng, Qian Wang

**Affiliations:** 1 Business School, Shandong Jianzhu University, Jinan, China; 2 School of Sport Communication and Information Technology, Shandong Sport University, Jinan, China; Hosei University: Hosei Daigaku, JAPAN

## Abstract

Innovation drive differs from investment drive and resource drive in that it focuses on knowledge and skills to promote productivity growth. By integrating technical standards within the framework of an innovation-driven development system in this work, theoretical implications for this development strategy may be revealed. Following our theoretical study, we built a PECM utilizing China’s inter-provincial panel data from 2007 to 2020 to investigate the long and short-term relationships between standardization, R&D, and innovation-driven development. The following are the key findings: First, both standardization and R&D are the nation’s critical engines of innovation-driven development. Second, standardization has the greatest impact on TFP through improving technical efficiency, whereas R&D drives both technical development and technical efficiency improvement. Third, while the influence of technical standard drafters’ production scale on scale efficiency was insignificant from 2007 to 2013, it became substantial after 2014 with China’s macroeconomic reform of "transforming the mode and changing the structure."

## 1. Introduction

Innovation is a critical driver of economic development, which distinguishes itself from growth that relies on resources and investments. Innovation-driven development is a dynamic process that involves gradually shifting from a "follower advantage" to an endogenous comparative advantage, driven by talent and knowledge to enhance productivity development [1–3]. However, the journey from innovation to increased production efficiency may involve a systematic evolutionary process. Furthermore, R&D alone could not trigger a catalytic effect on economic development. For instance, China’s massive and diverse multi-industrial economy has been growing steadily since the Reform and Opening and WTO accession, with GDP increasing from US$149.541 billion in 1987 to US$18.10 trillion in 2022. Unfortunately, economic growth has slowed significantly since the 2008 international financial crisis [4, 5]. Between 2007 and 2022, GDP growth decelerated from 14.23% to 3.00%, while the national patent grant growth rate increased from 17.11% in 2008 to 26.43% in 2021. The reasons behind this "scissors difference" in growth rates and how to ensure effective technology development as a driving force for social development [6] are crucial research questions.

We propose that technical standardization is a critical but understudied factor in this context. Standard adoption can significantly affect the behavior and outcomes of economic actors in the process of innovation generation and diffusion [7, 8]. At the micro and meso levels, standards establish the boundaries and connectivity of existing technology modules and facilitate the diffusion of technological innovation outputs through network externality mechanisms. Additionally, they signal future technological development paths and innovation directions in specific economies [9–12]. At the macro level, technical standards and technological innovation can complement each other and support national or regional economic growth [13, 14].

While past research has offered crucial implications for our study into the impact of technical standardization on innovation-driven growth], there is still scope for theoretical and empirical expansion in the theoretical mechanism framework and empirical analysis. We empirically investigate the impact of standardization on the overall innovation drive and its potential spatial or temporal variations between long and short-term as well as among geographic regions utilizing inter-provincial panel data for China from 2007 to 2020 based on a panel error correction model (PECM). The research is divided into four sections: the first is a theoretical explanation of the impact of technical standardization on innovation-driven development; the second is an empirical research design, which includes a description of econometric methods and relevant data; the third is an analysis of empirical results and further discussion; and the fourth is a conclusion and implications.

Possible theoretical contributions of this study might include: Firstly, we have included Technical Standardization in the theoretical framework of innovation-driven development, analyzing its impact on innovation-driven development from three dimensions: technological progress, technological efficiency, and economies of scale. This to some extent expands the existing literature on the economic effects of Technical Standardization. Secondly, we have used targeted measurement indicators in the design of the empirical research process, which can support our research content. Our empirical results provide practical evidence for exploring the impact of Technical Standardization on innovation-driven development. We hope that the research conclusions can contribute to the improvement of standardization and the effective implementation of innovation-driven development strategies in some countries or economies.

## 2. Theoretical considerations

### 2.1 Standard and technical standardization

Standards are important for fostering innovation and guiding progress. Standard dominance is often an indicator of a country’s innovation and competitiveness [15]. Standards also play a significant role in promoting sustainability and enhancing environmental performance at the societal level [16]. Incorporating standardized aspects into the theoretical framework of innovation-driven development can have theoretical implications for this approach [17]. Technical innovation is the most visible evidence of the innovation drive, and its R&D outputs are closely tied to the system of modern technical standards. These standards can be linked by standard essential patents (SEPs) and consist of mandatory requirements or guidance functions based on production technological activities, including technical requirements and specifications [18, 19].

Technical standardization is a process of developing technical standards aimed at establishing a common set of technical specifications and guidelines in specific fields. These standards not only guide technology development, production, and sales but also promote product interoperability, safety, communication, and sustainability [20–23]. Implementing technical standardization requires a series of processes such as development, implementation, supervision, and updates. Setting technical standards requires continuous research and testing based on relevant technology and market demands, which involves normative regulation of products and technology with representativeness. Furthermore, investment in technology standardization in various countries has been increasing with the internationalization of standardization continuing to strengthen [17, 24–26]. In this regard, standardization organizations play a crucial role in the development of technical standards, management of technical knowledge, and promotion of technical standards at various levels [27, 28]. The main functions of technology standardization are as follows: to promote technological innovation and improve its speed and quality, and to provide a fair and open platform for the market promotion of technology and its products [29]. At the same time, technology standardization can enhance the quality and safety of products and services, reduce manufacturing and trade costs, promote technological communication and exchange, and improve the efficiency of technical services [30].

Katz and Shapiro [31–33], Farrell and Saloner [34], Church and Gandal [35], and Shy [36] have established a sound theoretical foundation for investigating the role of technology standardization in innovation-driven economic growth. Following this, Blind and Jungmittag [37] used the Cobb-Douglas function to examine the key economic sectors of European nations and revealed that the stock of patents and the stock of technical standards might have a considerable effect on economic growth. Standards, according to Benjamin et al. [38], were an important issue in the innovation system. They concluded that standardizing institutions and infrastructures developed with government assistance may improve the industry’s business knowledge and technological capabilities. Firms which could run standardization and innovation would be able to gradually introduce high-quality standardized items into the market while progressing into more knowledge-intensive sectors in this situation. Jiang et al. [39] analyzed the ICT sector and discovered that technical standards and innovation seemed to be key variables influencing market competitiveness. This literature suggests that technical standardization might be critical for strengthening the industrial innovation system and increasing competitive advantage.

### 2.2 Technical standardization and innovation-driven development

**Fig 1:**
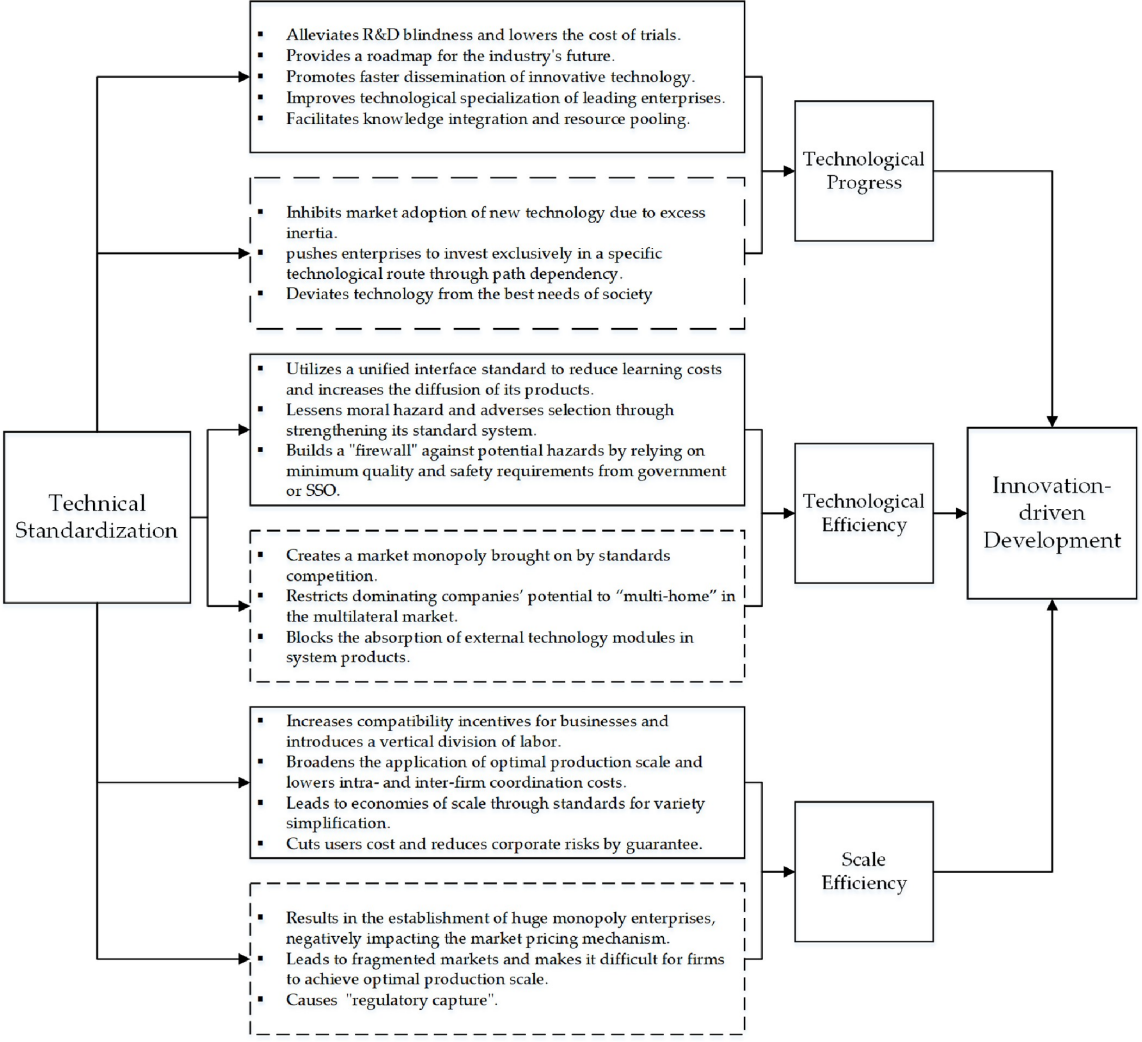
Mechanisms for the impact of technical standardization on innovation-driven development.

Total factor productivity is maybe the most commonly cited metric for innovation-driven development [40]. This econometric framework could decompose total factor productivity into technological progress, technological efficiency, and scale efficiency by calculating the DEA-Malmquist index, which corresponds to productivity gains from the frontier (reference technologies), best practice technologies, and economies of scale, respectively [41–43]. This indicator is being used to further analyze the mechanism underlying the influence of standardization on innovation-driven development, as shown in Fig 1.

#### 2.2.1 Standardization and technological progress

Technological advancement might have not just economic implications, but also social, political, cultural, ecological, and other multidimensional effects. Technological progress has long been recognized as an effective tool for achieving sustainable social development [44]. Based on various perspectives and time frames, it appears that most literature deduces that standardization has both positive and negative effects on technological progress, with one prevailing depending on how closely standardization activities match with technological progress in directions and time. Basic research, applied research, experimental development, industrial construction, market introduction, and technology dissemination may all occur in sequence. Technical standards that are well-matched might considerably hasten this procedure [7, 45]. The following are the primary causes. First, explicit and consistent technical standards would alleviate enterprises’ R&D blindness and lower the cost of trials. Standards define the "dominant design" of the items and give an implicit roadmap for the industry’s future. They may give enterprises reliable market foresight of new technologies as well as innovation incentives [46, 47].

Second, from the aspect of user selection bias, innovative technologies seem to be more likely to arise if market norms prevent new or incompatible technologies. As a result, significant savings in repetitious R&D activities would promote faster dissemination of innovative technology [48].

Third, enterprises incorporate their most recent R&D achievements into standards (standard essential patents, or SEPs), resulting in a "technology patenting—patent standardization—standard monopolization" cycle; this strategy has the potential to improve the technological specialization of leading enterprises [49–51]. To keep ahead of the competition, Qualcomm, for example, continued to set complex patents in mobile technological standards [52, 53].

Finally, standard alliances based on technological standards still can integrate knowledge. These collaborations might pool resources for large and essential technological research and development, as well as build an initial installed base. The formation of standards alliances might facilitate the entire process of R&D to market dissemination to realize the economic and social benefits of technological advancement [54–56].

Standardization, on the other hand, might inhibit technological growth owing to a variety of reasons such as technical features, user scales, and standard openness. First, Farrell and Saloner [34] proposed the idea of "excess inertia" from the standpoint of the installed base, stating that consumers may remain imprisoned by old technology while new technologies struggle to obtain market adoption. If new R&D achievements could not be economically rewarded by the market, technological developers would be deterred. Second, standardization’s "path dependency" [57] would push enterprises to invest more exclusively in a specific technological route. It becomes difficult for a single, homogeneous technological capability to innovate in an explorative manner and achieve significant technological advancements [58]. The likely cause of all of the above issues is that technical standardization deviates from socially optimal requirements.

#### 2.2.2 Standardization and technological efficiency

With exogenous technology assumption, the influence of standardization on the innovation impulse is primarily through institutional and organizational transformations. Compatibility and interface standards, which specify the method of connection inside and between industries [59], prepare the way for modular production from the producer’s standpoint. As a result, technical standards serve as "quasi-institutional frameworks" for internal collaborative partnerships. To decrease internal transaction costs, recurrent games among module makers are used to progressively establish rules, standards, and contracts. Simultaneously, modular network organizations are often governed by several parties led by technical leaders [60]. Through various alignments, intermediate product contracts and factor contracts are blended into a dynamic network of contracts with low transaction costs and high innovation capacity across companies and countries under the authority of industry standards or system rule makers [61]. A modular network organization’s integrator might maximize efficient contract networks by organizing various combinations of intermediate product contracts and factor contracts [62].

The dominating actor in modular production (the central signatory) alters the submodules and their suppliers continuously and dynamically so that the system product production model is endlessly near the ideal technology portfolio. Apple Inc., for example, constantly realigns its suppliers, pushing them to reflect its product plan. To showcase the iPhone 8’s user health management capabilities, Apple replaced several suppliers of motion sensor modules such as gyroscopes, accelerometers, and barometers in 2017. Although the rising complexity of product technology has exacerbated the information asymmetry between buyers and sellers, users can benefit from a more welcoming interaction environment and lower learning costs as a result of the unified interface standard. As a result, product diffusion might be sped up while the commercial potential of linked technology is completely realized. Additionally, by strengthening the standard system, moral hazard, and adverse selection may be lessened. Government or standardization organizations’ minimum quality and safety requirements could act as a "firewall" to lessen the market efficiency loss brought on by knowledge asymmetry while upholding their social responsibility to safeguard users’ welfare.

The market monopoly brought on by standards competition might be primarily to blame for the detrimental impact of standardization on technical efficiency. Although standards frequently contain a significant number of patents to represent the most recent technological advancements, the right to use them is typically not exclusive, making them "quasi-public commodities." As a result, businesses with incorporated core standards patents (SEPs) would rule the market. The dominating businesses could coordinate the cooperative network’s technical arrangements and interests. The nodal companies simultaneously build up exclusive interests in specific product modules as the cooperative production process progresses, which would restrict their potential to "muti-home" in the multilateral market. Thus, the dominant companies could be granted the right to "hold up," which unquestionably reduces the efficiency of market competition.

Additionally, member companies of a regular alliance typically have a high degree of interconnectedness. According to Keil [63], the development of technical standard alliances was a natural progression of business system competition. It would be difficult for the system’s products to enter the market if just one external technical innovation were not to be supported by the system as a whole, or if just one functional module were not to be adopted or compatible with the system’s products. However, to initiate a revolution, emerging technologies must offer 10 times the capability of the incumbent technologies, according to Andy Grove, the former CEO of Intel.

### 2.3 Standardization and scale efficiency

The Smith-Younger theorem shows that the degree of division of labor depends on market size, while market size depends on the level of division of labor, and thus the output will experience rising marginal rewards as the level of division of labor rises. Taylor (1911) acknowledged the importance of the division of labor in the organization’s economic success and then made it clear that the company should standardize its workers’ operating procedures, use standardized tools, machinery, and materials, and standardize the working environment. The vertical division of labor at the industrial level increases the incentive for businesses to be compatible [64]. Consistent technical standards would be required for the connection and interoperability of product modules, and their makers would establish a more reasonable division of work and relationship between them [65, 66].

Standardization is thus a prerequisite for achieving "internal economies of scale" and "external economies of scale," according to the division of labor. Compatible or interface standards, in particular, would lower intra- and inter-firm coordination costs and broaden the application of optimal production scale. Minimum standards for quality and safety would lower transaction and search costs for users while partially reducing moral hazard and adverse selection as a result of information asymmetry. This would help to expand the market scale, further the division of labor, and turn product production into a positive feedback loop of increasing returns to scale. Standards for variety simplification would directly lead to economies of scale by lowering unit prices and reducing the variety of products or even technologies by scaling raw material inputs, production scales, and distribution scales to the same standard [67]. Standards for information and testing would guarantee that the measured items are those that are "anticipated," saving users money (on selection, comparison, and testing) and reducing corporate risks (compensation, litigation). Swann [23] uses the ranking of gasoline to demonstrate how standards acceptance can lead to distribution economies of scale.

Nevertheless, from the standpoint of society or businesses, standardization’s effect on economies of scale might not even necessarily appear to be favorable. First, the adoption and competition of standards would necessitate the establishment of huge monopoly enterprises, which would negatively impact the market’s pricing mechanism. It might be a form of Marshall’s conundrum, which might cause social welfare to be lost. Second, there is a fundamental incompatibility between relatively constant technical standards and quickly shifting technology contexts and market demands. The investment in specialization could very well increase with the size of the production system developed using specific standards, which would reduce its ability to adapt to the environment [68, 69]. Third, with "regulatory capture", the aggregation of interests around large firms and standard dominant firms might also intervene in policy design and implementation by interfering with governments in nontransparent ways [23], to gain access to investment opportunities, tax incentives, financial subsidies, access to finance and government credit [70–72]. This appears to be especially common in countries lacking strong market mechanisms. Finally, to regulate monopolies, governments may allow competing standards to emerge concurrently when standardized production and standard competition lead to increased market concentration. In such cases, the market is likely to be fragmented, making it difficult for individual firms to achieve the optimal level of production scale.

## 3. Methods

### 3.1 Model

This study utilized two econometric models to investigate the relationship between standardization and innovation-driven development.

#### (1) DEA-Malmquist

The DEA-Malmquist is a method for evaluating the total factor productivity of companies, which combines the Data Envelopment Analysis (DEA) and the Malmquist index method. This method uses Data Envelopment Analysis to measure the efficiency level of companies or organizations under different output and input factors and measures the change in TFP by calculating the Malmquist index [73–76]. We use data envelopment analysis based on output-oriented variable returns to scale (VRS) to measure output distance and obtain total factor productivity by calculating the geometric mean of the Malmquist index before and after two periods to measure innovation-driven development. Malmquist [41] initially introduced the scale-factor theory and then used the ratio of the scale factor to construct the consumption quantity index. Following the concept of the Malmquist consumption index, Caves et al. [43] applied it to the analysis of production by constructing a productivity index from the ratio of distance functions and naming it the Malmquist productivity index in 1982. Related non-parametric methods have been widely used in recent years in the measurement of total factor productivity in China [77].

#### (2) Panel error correction model

The long-term and short-term effects of the interaction between the variables are investigated in this work, and the panel error correction model (PECM) is used with the consideration of the smoothness of the time series. According to Blackburne and Frank [78], our basic estimation model is set as follows:

$$\Delta y_{i,t}=\beta_{0}+\alpha {EC}_{i,t}+\sum_{j=1}^{2}\sum_{k=1}^{q}\beta_{i,j}{\Delta x}_{j,i,t-k+1}+\sum_{k=2}^{q}\beta_{i}{\Delta y}_{i,t-k+1}+\varepsilon_{i,t}$$ (1)

In Eq (1), *EC* is the error correction item with *Δy*_*i*,*t*_ as the dependent variable, and *Δx* as the independent variable; *j* is the number of independent variables, there are three involved in this, *tfpch*, *standard*, and *patent*. *k* is the lag number for each item and *q* is the best lag number of the model due to information criterion; *i* is the number of decision-making units (DMUs) and *t* is the periods; *α* is the coefficient of the error correction item, which is used to indicate the degree and direction of correction of the long-term covariance on the dependent variable (the internal coefficients are *λ* and *θ*, denoting the coefficients of the two independent variables within the error correction formula, respectively); *β* is the coefficient of the short-term effect of the independent variable on the dependent variable, and *ε* is the residual item.

### 3.2 Variables and data

#### (1) Variables

Total factor productivity (TFP) at the province level in China was used to gauge each region’s amount of innovation-driven growth. Total Factor Productivity (TFP) measures the true impact of an innovation-driven economy’s growth by referring to the rise in production brought about by technical advancement and capability realization in addition to each factor’s input (mostly labor and capital) [79]. The GDP data of 31 provinces, autonomous regions, and municipalities directly under the Central Government of China (Hong Kong SAR, Macau SAR, and Taiwan Province were not included here due to data availability) are used as output variables, the number of employed persons in each region in each year as labor input variables, and the fixed capital stock as capital input variables are calculated using the data envelopment analysis method. According to Färe et al. [42], total factor productivity (*tfpch*) was further decomposed into technical advancement (*techch*), pure technical efficiency (*pech*), and scale efficiency (*sech*) under the premise of variable returns to scale (VRS).

As a measure of the degree of technical standardization, we used the logarithm of each province’s standard development contribution index (standard) in each period. The standard development contribution index is calculated by adding the contribution values of all entities involved in standard development during a given period. The contribution value of the drafting entities is calculated by adding their standard contribution scores in each standard draft based on their ranking. The standard contribution index takes into account not only the number of times the regions participate in standard drafting but also their contribution to the process. It is determined as follows:

$${standard}_{it}=ln(\sum_{s=1}^{m_{i,t}}\sum_{r=1}^{n_{s,t}}{score}_{r,s,t})$$ (2)

In Eq (2), *standard*_*i*,*t*_ is the standard contribution index of region *i*; *m*_*i*,*t*_ is the number of entities that participated in standard development of region *i*; *n*_*s*,t_ is the number of standards in which draft entity *s* participated; and *score*_*r*,*s*,*t*_ *i*s the contribution score of entity *s* in its *r*th standard in period *t*. Following the guidelines of the China National Standards Literature Sharing Service Platform (https://www.nssi.org.cn/nssi/front/index.jsp), the first place in the list of the specific standard application takes 1 point, while the second to fifth places take 0.8, 0.6, 0.4 and 0.2 points respectively, and 0.1 points after the sixth place.

Besides this, considering the relationship between technological assets and technical standardization, we introduced the technological R&D variable (*patent*) to alleviate the omitted variable bias and effectively separate the impact of standardization on total factor productivity. The logarithm of the annual number of patents granted in each region was used to approximate patents.

#### (2) Data

For the period 2007–2020, we used panel data from 31 provinces, autonomous regions, and municipalities directly under the Central Government of China (Hong Kong SAR, Macau SAR, and Taiwan Province were not included here due to data availability). GDP data was used for output in total factor productivity calculations, and the GDP index deflator was used for the year 2000 as the base period. The number of employed people in each region during the calendar year is used to calculate labor. The perpetual inventory method is used to compute the capital stock, with the fixed capital stock in 1997 serving as the base period stock data and the flow data using total fixed capital formation in each year. We set the annual depreciation rate at 9.6% and deflate the capital stock in each region based on the fixed capital price index [80].

The economic data for this study came from the statistical annals of China’s regions, the Compilation of Statistics for 60 Years of New China, and other sources. The standard contribution value stats retrieved from the "National Standard Literature Sharing Service Platform—National Standard Drafting Unit Big Data Platform" (http://bigdata.nssi.org.cn/), which is managed by the General Administration of Quality Supervision, Inspection, and Quarantine of China and the National Standards Committee and built and operated by the China National Institute of Standardization’s National Standards Library. The China Statistical Annals (http://www.stats.gov.cn/) and the China Stock Market and Accounting Research Database (CSMAR) were used to obtain patent data.

## 4. Results

### 4.1 Descriptive statistics

**Fig 2:**
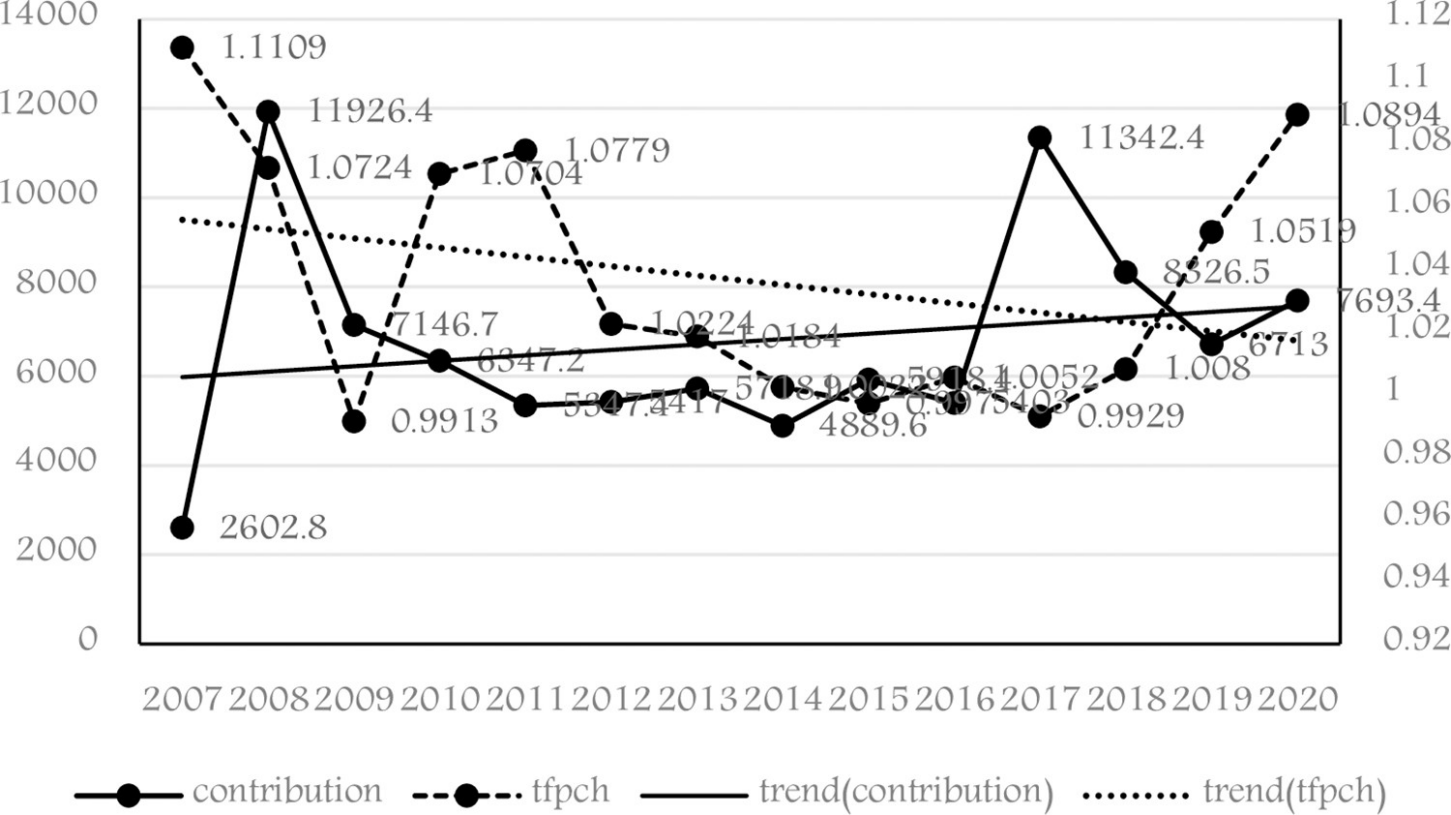
China’s nationwide TFP and standard contribution index, during 2007–2020.

Based on the previous part, we first estimated the national and regional TFP for 2007–2020, as shown in Fig 2, and then explored the national standard contribution index for this time. To begin, TFP has an annual change of 0.9984, with the highest number coming in 2007. (1.1109). Because of the 2008 financial crisis, the TFP declined substantially in 2009 to its lowest level (0.9913) during the research period. TFP’s general trend ranges downward and then upward from 2010 to 2020, forming a "V" shape.

The national total standards contribution index rose dramatically and peaked in 2008. The fundamental cause for this appears to be a change in the legislative framework. The Standardization Law of the People’s Republic of China was updated in November 2007 and proclaimed on January 1, 2008. The redesign included a new standard system, management system, and operating mechanism, which prompted the upgrade of a large number of industry standards to national standards. Concurrently, the revision sparked widespread societal concern and encouraged social entities to join in standardizing operations. Fig 1 also shows that the standard contribution index began to fall after 2008, then steadily steadied until rising strongly again in 2017.

The trend lines in Fig 1 show that the TFP falls significantly during the study period, while the standard contribution index rises significantly. The trend lines of the two series show a clear "scissor difference," implying that they may have an inverse correlation. Table 1 displays the descriptive statistics for the panel data. Total productivity and standard contribution value or technology R&D have positive but insignificant correlation coefficients. At the 1% level, the correlation between the value of standard contribution and technological R&D is positive. Given that the economic effects of technical standards (particularly national standards) are ongoing processes, we may need to investigate both their short-term and long-term effects in our research [8, 81].

**Table 1:** Descriptive statistics of the provincial panel data.

Variable	Obs	Mean	S. D.	Min	Max	Pearson’s correlation coefficient		
						*tfpch*	*standard*	*patent*
*tfpch*	434.0000	1.0056	0.0325	0.8820	1.1365	1.0000		
*standard*	434.0000	4.4226	1.4968	-1.6094	8.1088	0.0272	1.0000	
*patent*	434.0000	9.5590	1.7306	4.2195	13.4726	0.0202	0.7879***	1.0000

Note: *, **, *** indicate significance at the 10%, 5%, and 1% levels, respectively. Hereinafter.

### 4.2 Construction and regression results of the Panel Error Correction Model (PECM)

We ran unit root tests on each variable before building the PECM. Given the possibility of cross-sectional correlation among data due to economic activity interactions and spillover effects between regions, we used Breitung’s [82] approach for the unit root test. Tests for the three horizontal values of the variables, as shown in Table 2, showed that both *tfpch* and *patent* have the unit root, but their respective 1-way differences all passed the test at the 1% level. As a result, *standard* is an *I(0)* series, whereas *tfpch* and *patent* are both *I(1)* series.

**Table 2:** Breitung unit-root test.

Variable	Statistic	p	Stationary(p<0.1)
*tfpch*	0.2274	0.5899	no
*standard*	-3.8628	0.0001	yes
*patent*	11.6773	1.0000	no
*D*.*tfpch*	-5.7148	0.0000	yes
*D*.*standard*	-3.7430	0.0001	yes
*D*.*patent*	-9.4214	0.0000	yes

We tested the cointegration with the Westerlund [83] method. We investigated the existence of homogeneous panel cointegration and heterogeneous panel cointegration using one variable as the dependent variable and the other two variables as independent variables. As shown in Table 3, the null hypotheses (H0) were all rejected at the 1% significance level in the homogeneous panel cointegration test, indicating the existence of cointegration relationships for all individuals. Similarly, the null hypotheses were all rejected at the 1% significance level in the heterogeneous panel cointegration test, indicating the existence of cointegration relationships for all individuals.

**Table 3:** Westerlund test for cointegration.

Dependent Variable	H0: No cointegration H1a: All panels are cointegrated		H0: No cointegration H1b: Some panels are cointegrated	
	Statistic	p	Statistic	p
*tfpch*	-2.4003	0.0082	-2.6268	0.0043
*standard*	-3.5948	0.0002	-4.6946	0.0000
*patent*	5.8355	0.0000	12.9616	0.0000

To find the best lag order for the PECM, we first created the autoregressive distributed lag model *ARDL (q1*, *q2*, *q3)*, where q represents the lag order of each variable (1≤*q*≤6). The optimal lag order for all three variables was identified as 1 according to the AIC and BIC criteria after estimating all 216 regression equations. Table 4 shows the regression results of the PECM with *tfpch*, *standard*, and *patent* as dependent variables (from data from all provinces in China, including the eastern, central, and western provinces).

**Table 4:** The regression results of PECM I^#^.

		nationwide			eastern			central			western		
DV		*Δtfpch*	*Δstandard*	*Δpatent*	*Δtfpch*	*Δstandard*	*Δpatent*	*Δtfpch*	*Δstandard*	*Δpatent*	*Δtfpch*	*Δstandard*	*Δpatent*
EC	*standard*	0.0398***		1.4627***	0.0129***		1.2297***	0.1105***		0.9388*	0.0883***		2.3058
		(0.0065)		(0.2588)	(0.0043)		(0.3109)	(0.0285)		(0.4867)	(0.0162)		(2.0390)
	*patent*	0.0168***	-0.0782***		-0.0196***	0.2280***		0.0647***	-0.2148***		0.0201***	-0.0695***	
		(0.0018)	(0.0169)		(0.0017)	(0.0680)		(0.0120)	(0.0453)		(0.0017)	(0.0227)	
	*tfpch*		2.5022***	-17.3759***		7.6080***	-21.9328***		4.5103***	1.2797		2.2554**	0.9134
			(0.6942)	(3.5091)		(2.5122)	(3.9311)		(1.3634)	(8.1635)		(1.0132)	(17.9981)
ECM	*ec*	-0.3546***	-1.0537***	-0.0736***	-0.7145***	-0.9222***	-0.1202***	-0.1725*	-1.1416***	-0.0684*	-0.1851***	-1.1097***	-0.0394
		(0.0656)	(0.0607)	(0.0164)	(0.1066)	(0.1073)	(0.0377)	(0.0923)	(0.0740)	(0.0377)	(0.0697)	(0.1222)	(0.0243)
	*Δstandard*	-0.0118***		-0.1012***	-0.0123**		-0.1380***	-0.0036		-0.0738*	-0.0142***		-0.0830**
		(0.0026)		(0.0217)	(0.0052)		(0.0343)	(0.0038)		(0.0398)	(0.0047)		(0.0356)
	*Δpatent*	-0.0184**	0.1138		0.0068	-0.1526		-0.0300*	0.4378*		-0.0251	0.1615	
		(0.0081)	(0.1418)		(0.0112)	(0.2256)		(0.0172)	(0.2404)		(0.0154)	(0.2682)	
	*Δtfpch*		-3.7437**	-0.4186		-7.4174***	-1.9462		-2.9999*	0.1592		-3.7919	-0.5742
			(1.6394)	(0.8449)		(1.8423)	(1.5024)		(1.6353)	(0.6510)		(3.7285)	(0.7952)
	*Constant*	0.2330***	2.7766***	1.6997***	0.8207***	-4.0556***	3.3204***	-0.0091	2.1819***	0.5136***	0.0855**	1.9294***	0.1921
		(0.0452)	(0.3046)	(0.3337)	(0.1240)	(0.4461)	(0.9852)	(0.0103)	(0.2121)	(0.1747)	(0.0416)	(0.4682)	(0.7545)
Observations		403	403	403	143	143	143	104	104	104	156	156	——
Method		*pmg*	*pmg*	*pmg*	*pmg*	*pmg*	*pmg*	*pmg*	*pmg*	*pmg*	*pmg*	*pmg*	*dfe* ^##^

Notes: #. *, **, *** indicate significant at the 10%, 5%, and 1% levels, respectively. The EC section reported the estimated values of each variable of the error correction term, and the ECM section reported the estimated coefficients of the error correction model. The standard deviations were given in parentheses. We preferred pmg mixed group mean estimates that equalized the cross-sectional coefficients in the error correction term. intergroup heterogeneity accounted for the short-term adjustment.

##. Note: In cases where "initial replication failure" or circular iterations occurred during estimation, the dfe group average estimation method was used [78].

The estimation results of the error correction term (*EC*) in the estimated equation with *Δtfpch* as the dependent variable revealed that both *standard* (*β =* 0.0398; *p<*0.01) and *patent* (*β =* 0.0168; *p<*0.01) have a long-term significant positive effect on *Δtfpch*. This suggested that standardization could help TFP. The rise in R&D patents provides a steady and long-term source of fuel for innovation-driven development. The coefficient of the error correction term *ec* was significantly negative (*β =* -0.3546; *p<*0.01) in the error correction model (ECM), indicating the existence of a reverse correction mechanism—when TFP deviates from the long-run equilibrium relationship by 1 unit, it is adversely corrected by approximately 35% to ensure systemic stability. The ECM equation’s *Δstandard* lag term (*β =* -0.0118; *p<*0.01) and *Δpatent* lag term (*β* = -0.0184; *p<*0.05) coefficients are both significantly negative, suggesting that standardization and R&D short-circuit innovation-driven development. The first one can follow from the introduction of new standards that altered the climate of competition.

From this, it follows that, in the long term, standardization and R&D patents can be complementary strategies for firms seeking to improve their TFP. While standardization is a method to reduce costs and improve efficiency, R&D patents can provide firms with a competitive advantage through innovative products or processes. During the standardization process, firms can identify areas that require innovation and invest in R&D to strengthen their patent portfolio. This combination of strategies can drive productivity and efficiency, reducing costs in the long term and improving a firm’s ability to innovate and adapt to changing market conditions. In the short term, there may be a tradeoff between standardization, R&D, and innovation-driven development. The resources allocated to standardization and R&D may come at the expense of other activities that are critical to driving innovation. Additionally, excessive focus on standardization may discourage experimentation and creativity, which are necessary for innovation. Similarly, over-investment in R&D may lead to an over-reliance on technology and a lack of focus on customer needs. The R&D’s temporary negative effect on TFP might be the result of provincial governments’ funding policies for certain patents, which distorted the motivation of R&D entities to apply for patents. This could lead to the "lock-in" of technology products in the short term and affect the appearance and application of new technologies. Thereafter, a great number of inferior patents could be produced. Despite the national patent inspection and approval procedure, which would lessen this adverse effect, these patents would still not be helpful for TFP growth and their inclusion in SEPs might diminish the contribution of R&D to TFP.

The error correction term (EC) estimation results in the equation with *Δstandard* as the dependent variable showed that *patent* (*β =* -0.0782; *p<*0.01) had a significant positive effect on *Δstandard* in the long run, while *tfpch* (*β =* 2.5022; *p<*0.01) had a significant negative effect. This revealed that R&D achievements have contributed to the availability of technical resources for the standardization process, while TFP growth has reinforced the adoption of established standards. However, these effects can result in lock-in effects, which may discourage the development of new standards. Such lock-in effects occur when existing standards become firmly established and difficult to replace, in part due to the high costs associated with changing them. Finding a balance between the benefits of standardization and the need for encouraging new technological developments is therefore crucial to promote ongoing progress and maintaining a dynamic business environment.

The *Δtfpch*’s lag term coefficients (*β =* -3.7437; *p<*0.05) were all statistically negative, showing that TFP has a detrimental influence on standardization in the short run. One potential reason for this is that lock-in effects created by existing standards may limit the development of new ones, but as productivity levels increase, the market demands higher levels of technological advancement and innovation for standards. This conflict between adhering to established standards and the need for new ones could potentially delay the standardization process.

The results of the error correction term (*EC*) estimation in the equation with *Δpatent* as the dependent variable demonstrated that *standard* (*β =* 1.4627; *p<*0.01) had a significant positive long-term influence on *Δpatent*, but *tfpch* (*β =* -17.3759; *p<*0.01) seemed to have a significant negative long-term effect. The error correction term *ec* coefficient in the error correction model (ECM) was significantly negative (*β =* -0.0736; *p<*0.01), suggesting that a reverse correction process was present. The coefficient of the *Δtfpch* lag term (*β =* -0.418; *p>*0.1) was negative but not significant in the ECM equation, whereas the *Δstandard* lag term (*β =* -0.1012; *p<*0.01) used a significant negative impact on the *patent*. This meant that, despite its restrictive effect on technology development in the near term, standardization might have the potential to enhance new technology created in the long run. Certain standardized, particular technology approaches might be less appealing than competing technology paths. However, if a definite technological direction is established, the new technology R&D process would be more efficient. TFP seems to be a direct result of technological advancement. TFP growth may result from cost reductions or profit increases, but it is also likely to encourage industry monopolization. The patent abuses and the hostile acquisitions due to monopoly positions could be serious obstacles to technological development [84, 85].

The results of the sub-regional regression revealed that the effect of standardization (β = 0.0129; p<0.01) on TFP in the Δ*tfpch* equation for the eastern provinces was broadly consistent with the national situation, whereas the long-term effect of technology R&D (*β* = -0.0196; *p*<0.01) was significantly negative and the short-term effect was not significant. One possible explanation for this effect could be related to the time needed to effectively implement new technology, which may be delayed due to factors such as the diffusion of knowledge and skill development. Another possible explanation relates to the dynamic nature of technology and the potential for newly developed technologies to become obsolete quickly, reducing their capacity to drive long-term growth. However, regardless of the cause of the negative long-term effect, these findings highlight the importance of considering both short and long-term effects when assessing the impact of technology R&D on economic growth. Policymakers should also consider the potential challenges associated with implementing new technologies and the conditions that are necessary for their success, such as investment in education and infrastructure. Short-term relationships between variables in the central provinces were essentially similar to national ones: standardization (*β =* 0.1105; *p<*0.01) and R&D (*β =* 0.0647; *p<*0.01) both contributed to TFP as the two engines of innovation-driven development. A significant inverse adjustment of the error correction term (*β =* -0.1725; *p>*0.1) indicated a long-term stable relationship. The short-run negative effect of standardization on TFP (*β =* -0.0036; *p>*0.1) was, however, insignificant. The regression results for western provinces highlighted that standardization (*β =* 0.0883; *p<*0.1) and R&D (*β =* 0.0201; *p<*0.1) might have significantly positive long-run influences on TFP, which would be consistent results.

### 4.3 Robustness

We used the DEA-Malmquist method to calculate total factor productivity as a measure of innovation-driven development. However, this method has been criticized for being sensitive to the depreciation rates of fixed assets, technical assumptions, and extreme values. Therefore, we conducted robustness tests on the main results from four perspectives. Firstly, we used an average depreciation rate of 12.3% for all capital stock, based on the depreciation rates of physical capital stock and research and development capital stock of 9.6% and 15% [77], respectively (Method1). Secondly, we relaxed the strong assumptions of sequential production technology and sustained economic growth and changed the output model to an input model in the DEA-Malmquist calculation (Method2). Thirdly, we trimmed 1% of the extreme values in the data and conducted regression analysis on the new dataset (Method 3). Fourthly, we used the quantity of dominant and leading technology standards to measure Technical Standardization instead of the standard contribution index and incorporated the new variable into the panel error correction model (Method4). The robustness test results are shown in Table 5, which indicates that the coefficients of the main indicators remained directions (positive or negative) and significant, demonstrating the robustness of our main conclusions regarding the relationship among total factor productivity, Technical Standardization, and technology research and development.

**Table 5:** The result of the robust test.

		Method1: Adjusting depreciation rate for DEA			Method2: Adjusting Technology for DEA			Method3: Truncating the variables			Method4: Substituting the variable of standardization		
DV		*Δtfpch*	*Δstandard*	*Δpatent*	*Δtfpch*	*Δstandard*	*Δpatent*	*Δtfpch*	*Δstandard*	*Δpatent*	*Δtfpch*	*Δstandard*	*Δpatent*
EC	*standard*	0.0348***		1.4738***	0.0342***		1.4692***	0.0354***		1.6818***	0.0321***		1.4919***
		(0.0052)		(0.2458)	(0.0052)		(0.2458)	(0.0052)		(0.2197)	(0.0051)		(0.2920)
	*patent*	0.0127***	-0.0862***		0.0127***	-0.0874***		0.0143***	-0.0889***		0.0158***	-0.1821***	
		(0.0020)	(0.0166)		(0.0021)	(0.0165)		(0.0020)	(0.0166)		(0.0021)	(0.0149)	
	*tfpch*		2.5924***	-17.3609***		2.6394***	-17.3968***		2.6770***	-18.1908***		2.3852***	-20.8036***
			(0.6738)	(3.4682)		(0.6660)	(3.4716)		(0.6667)	(3.2318)		(0.6433)	(3.7638)
ECM	*ec*	-0.3816***	-1.0427***	-0.0741***	-0.3869***	-1.0419***	-0.0737***	-0.3817***	-1.0471***	-0.0720***	-0.4179***	-1.0394***	-0.0643***
		(0.0697)	(0.0566)	(0.0164)	(0.0701)	(0.0565)	(0.0164)	(0.0692)	(0.0563)	(0.0161)	(0.0649)	(0.0577)	(0.0155)
	*Δstandard*	-0.0116***		-0.1037***	-0.0116***		-0.1031***	-0.0113***		-0.1045***	-0.0111***		-0.1002***
		(0.0025)		(0.0221)	(0.0025)		(0.0220)	(0.0025)		(0.0221)	(0.0024)		(0.0209)
	*Δpatent*	-0.0181**	0.0980		-0.0183**	0.0981		-0.0155*	0.1113		-0.0193**	0.1220	
		(0.0079)	(0.1429)		(0.0079)	(0.1424)		(0.0083)	(0.1450)		(0.0077)	(0.1546)	
	*Δtfpch*		-3.9035**	-0.4819		-3.8893**	-0.4956		-3.7139**	0.0437		-2.5601*	-0.6124
			(1.6549)	(0.8938)		(1.6030)	(0.8771)		(1.6259)	(0.8354)		(1.4285)	(0.7984)
	*Constant*	0.2738***	2.7627***	1.7007***	0.2787***	2.7235***	1.6984***	0.2665***	2.7216***	1.6470***	0.2978***	3.8656***	1.7206***
		(0.0521)	(0.3052)	(0.3331)	(0.0525)	(0.3047)	(0.3336)	(0.0503)	(0.3093)	(0.3242)	(0.0461)	(0.3409)	(0.3664)
Observations		403	403	403	403	403	403	403	403	403	403	403	403
Method		*pmg*	*pmg*	*pmg*	*pmg*	*pmg*	*pmg*	*pmg*	*pmg*	*pmg*	*pmg*	*pmg*	*pmg*

## 5. Discussion

TFP could be decomposed into technological advancement and technical efficiency (which is further decomposed into pure technical efficiency and scale efficiency according to the FGNZ model). These components were included as dependent variables in the PECM, and the results are reported in Table 5. Because the term *ec* in all models had a 1% reverse correction impact, the variables in the system showed stable correlations in the long run.

The first section of Table 6 offered the regression results of the PECM, consisting of *techch*, *standard*, and *patent*. In the equation with *Δtechch* as the dependent variable, the long-term effects of standardization and technological R&D on technological progress were significantly positive, while the short-term effects were negative. Using *Δstandard* as the dependent variable, the long-run effect of technology R&D on standardization was significantly negative, whereas technological progress was not significant. With the dependent variable *Δpatent*, the long-term effect of standardization on technological development was significantly positive, while that of technological progress was significantly negative.

**Table 6:** The regression results of PECM II.

*component =*		*techch*			*effch*			*pech*			*sech*		
DV		*Δtechch*	*Δstandard*	*Δpatent*	*Δeffch*	*Δstandard*	*Δpatent*	*Δpech*	*Δstandard*	*Δpatent*	*Δsech*	*Δstandard*	*Δpatent*
EC	*standard*	0.0398***		1.4627***	0.0068***		0.5178*	0.0240***		1.4612***	-0.0008		0.5467*
		(0.0065)		(0.2588)	(0.0019)		(0.2806)	(0.0030)		(0.2417)	(0.0066)		(0.2818)
	*patent*	0.0168***	-0.0782***		0.0078***	0.0161		-0.0114***	-0.0611***		0.0073***	-0.0605***	
		(0.0018)	(0.0169)		(0.0008)	(0.0302)		(0.0014)	(0.0222)		(0.0025)	(0.0216)	
	*component*		2.5022***	-17.3759***		2.3939	28.4113***		0.2140	-24.3095***		1.6046*	17.4739***
			(0.6942)	(3.5091)		(1.7805)	(8.8420)		(0.8213)	(6.4454)		(0.8477)	(4.2579)
ECM	*ec*	-0.3546***	-1.0537***	-0.0736***	-0.4441***	-0.9595***	-0.0745***	-0.4599***	-1.1157***	-0.0808***	-0.6324***	-1.1135***	-0.0729***
		(0.0656)	(0.0607)	(0.0164)	(0.0224)	(0.0494)	(0.0163)	(0.0847)	(0.0409)	(0.0178)	(0.0496)	(0.0489)	(0.0179)
	*Δstandard*	-0.0118***		-0.1012***	-0.0054***		-0.0427**	-0.0068***		-0.1081***	-0.0005		-0.0831***
		(0.0026)		(0.0217)	(0.0009)		(0.0210)	(0.0023)		(0.0227)	(0.0024)		(0.0173)
	*Δpatent*	-0.0184**	0.1138		0.0100**	0.1039		-0.0210***	0.1300		-0.0118*	0.0629	
		(0.0081)	(0.1418)		(0.0040)	(0.1277)		(0.0068)	(0.1310)		(0.0062)	(0.1289)	
	*Δcomponent*		-3.7437**	-0.4186		-13.2459***	2.8124*		-0.7270	-1.0134		-0.5278	-6.2668
			(1.6394)	(0.8449)		(2.3938)	(1.5127)		(1.5085)	(0.8370)		(2.3511)	(4.2611)
	*Constant*	0.2330***	2.7766***	1.6997***	0.3966***	1.7329***	-1.3581***	0.4613***	5.3529***	2.4038***	0.5890***	3.7469***	-0.5113***
		(0.0452)	(0.3046)	(0.3337)	(0.0197)	(0.2750)	(0.3516)	(0.0838)	(0.3376)	(0.4890)	(0.0496)	(0.3138)	(0.1757)
Observations		403	403	403	403	403	403	403	403	403	——	403	403
Method		*pmg*	*pmg*	*pmg*	*pmg*	*pmg*	*pmg*	*pmg*	*pmg*	*pmg*	*dfe*	*pmg*	*pmg*

Note: In cases where “initial replication failure” or circular iterations occurred during estimation, the dfe group average estimation method was used.

These results demonstrated that the twin wheels of standardization and technical R&D might generate technological progress, one of the major elements of innovation-driven development. Standardization plays a crucial role in reducing the complexity of technological development and improving its diffusion. It facilitates the transfer of knowledge and technologies across different domains, industries, and countries. Additionally, standards provide a common language and framework that can be used to improve the interoperability and compatibility of different technologies, which increases their adoption and use. At the same time, technical R&D is essential for technological progress. It enables the creation and improvement of new and innovative technologies, which can enhance productivity, create new products and services, and promote economic growth. The combination of standardization and technical R&D can generate a virtuous circle that leads to sustained technological progress and economic development. Changes in the production technology frontier are caused by technological advancement, reflecting an internal reorganization of the “techno-economic” framework. Meanwhile, emerging standards tend to “challenge” the existing dominating designs [86–88].

The second section presented the PECM regression results on *effch*, *standard*, and *patent*. With *Δeffch* as the dependent variable, the long-term effects of standardization and R&D on technical efficiency were both significantly positive, the short-term effects of standardization were significantly negative, and the short-term effects of technical R&D were significantly positive. The long-term effects of both standardization and R&D on technical efficiency are significantly positive. A thorough analysis of the data demonstrated that standardization and technical R&D might both accelerate the rise of technological advancement and efficiency in the long term, acting as twin drivers of TFP. Standardization reduces complexity and improves diffusion, while R&D creates and improves new technologies. Both help increase productivity, create new products and services, and promote economic growth. The positive effects of standardization and R&D on technical efficiency are long-lasting and contribute to sustained economic development.

China’s development process provides evidence of this view. Before the twenty-first century, China had been reforming and opening up for two decades, but there were still wide gaps in economic progress when compared to wealthy countries. Many Chinese industrial sectors were still plagued by poor productivity technologies and had only recently attained their current levels [89, 90]. China’s further reform and opening up, WTO accession and the information technology revolution have enabled the country to make rapid technological advances in major industrial sectors since 2000 [91, 92]. China has always wanted to enhance technological efficiency along with technological progress. There are two major strategies for promoting TFP. The first is to achieve technological absorption and upgrading through FDI from multinational firms in China, and the second is to achieve technological breakthroughs and marketable applications by domestic Chinese firms through independent R&D [93, 94].

The third and fourth parts were the results of the regression of PECM consisting of *pech* and *sech* with other variables, respectively. We focused on analyzing the regression results on *sech*, *standard*, and *patent*. With *Δsech* as the dependent variable, the long- and short-term effects of standardization on scale efficiency were insignificantly negative, while the long-term effect of technology development was significantly positive and the short-term effect was significantly negative. When *Δsandard* was the dependent variable, the long-term effect of R&D on standardization was significantly negative, while the short-term effect was insignificantly positive. The long-term effect of scale efficiency on standardization was significantly positive, and the short-term effect was insignificantly negative. When *Δpatent* served as the dependent variable, the long-term effect of standardization on R&D was significantly positive, while the short-term effect was significantly negative; the long-term effect of scale efficiency was significantly positive, while the short-term effect was insignificantly negative.

**Fig 3:**
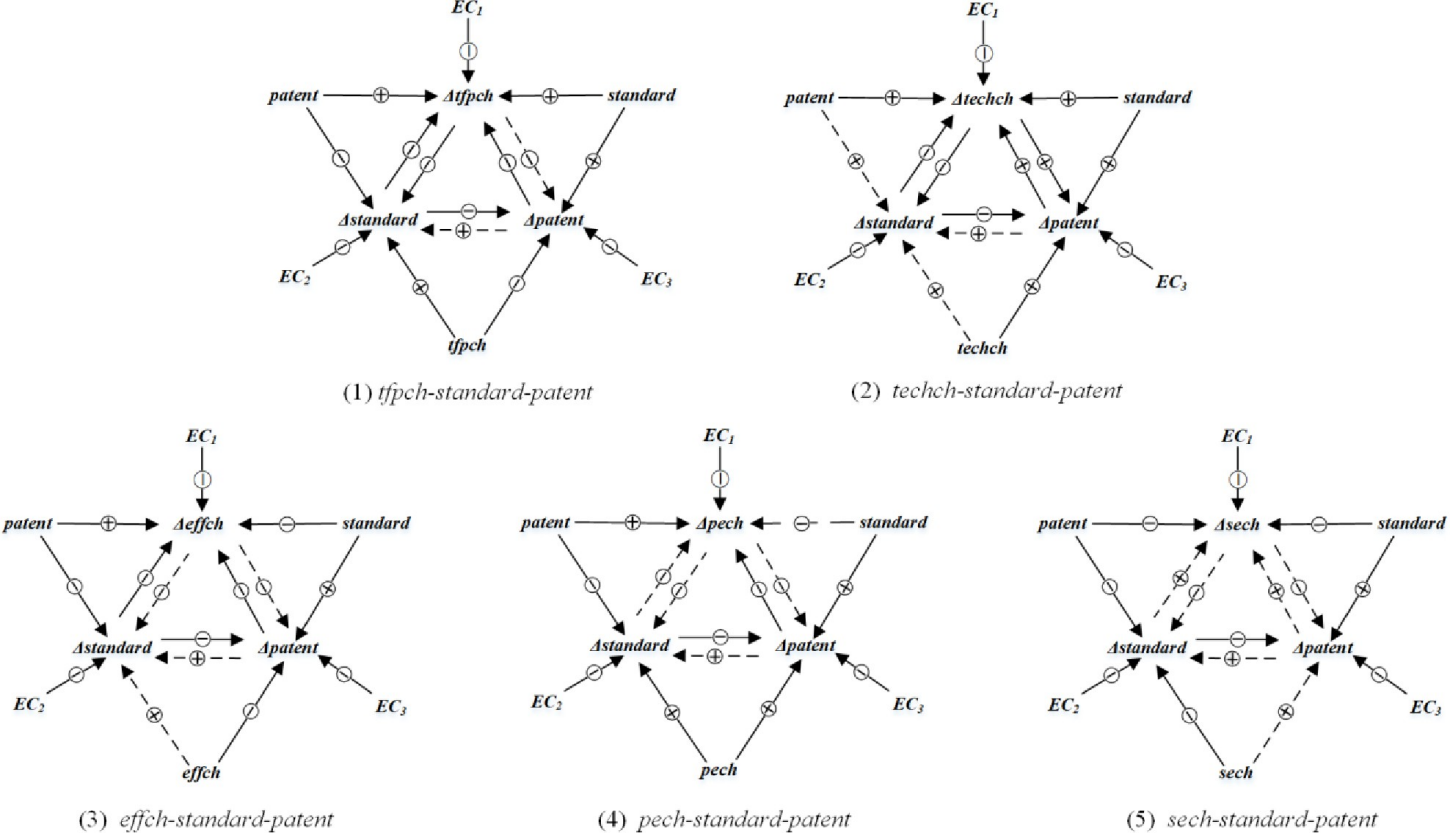
Summary diagram of the relationship between variables in the PECM. Note: The solid (dashed) arrows indicate significant (insignificant) effects, and the circles "+" and "-" indicate positive and negative effects, respectively.

Based on the PECM, relationships between the provincial TFP’s components, standardization, and technology R&D were summarized in Fig 3.

It is worthwhile to learn about the relationship between standardization and scale efficiency. As previously stated, there was a logical link between the two, with standardization being one of the key conditions for obtaining scale rewards, and incremental scale rewards reinforcing the use of production standards [14, 95]. If this is the case, why was the effect of standardization on scale efficiency insignificant in this case? The main reason, we believed, was that the standardization measurement above did not accurately reflect the scale factor. The main reason, we believe, is that the standardization measurement does not accurately reflect the scale factor. In terms of contribution, two primary factors influenced standardization for a specific province: the total number of standards drafted by its entities and the entities’ status during the drafting process (leading, hosting, or participating). High levels of the first factor would imply that more standards would emerge across the board. As a result, the single standard would be less influential in general; the second element would have more influence; as a result, they would tend to adopt their dominant standards. In this case, standards-based competition might become more fierce, potentially reinforcing monopolies’ deteriorating market efficiency.

To deal with this issue, we attempted to design a “drafter production scale logarithm” (*ln_scale_drafter*) as a proxy for *standard*. Logically, standard-drafters should be the most active promoters and adopters of standardization in each region. The economic impact of standards would be visually evident if the increase in the production scale of these entities could generate significant scale efficiency gains for the local region. *ln_scale_drafter* is calculated as follows.


$${ln\_scale\_drafter}_{it}=ln(\frac{{GDP\_drafter}_{it}}{{Num\_drafter}_{it}})$$ (3)

*Num_drafter*_*it*_ in Formula (3) is the number of standards drafted in region *i* during period *t*. The data came from the "National Standard Drafting Unit Big Data Platform" (http://bigdata.cssn.net.cn). *GDP_drafter*_*it*_ is the total economic output of standard drafting entities in region *i* in period *t*, which was estimated based on the GDP and the number of legal entities in each region. The estimation steps are:

Step 1: Dividing the total economic production into two parts, respectively generated by standard drafting entities (*Num_drafter*) and other entities (*Num_other*). *GDP*_*it*_ = *α*_*i1∙*_*Num_drafter*_*it*_^*βi1*^+*α*_*i2∙*_*Num_other*_*it*_^*βi2*^ is the total regional output form based on the Cobb-Douglas production function.

Step 2: For each region, *i*, estimate its *α*_*i1*_, *α*_*i2*_, and *β*_*i1*_, *β*_*i2*_ respectively using nonlinear regression. Output is obtained from GDP data, from the Chinese Statistical Annual.

Step 3, calculating the "drafter production scale logarithm", the formula is *GDP_drafter*_*it*_ = *α*_*i1∙*_*Num_drafter*_*it*_^*βi1*^.

We included *ln_scale_drafter* as a proxy for *standard* in the PECM. The stationarity test showed that it was *I(1)*. The Westerlund cointegration test demonstrated the existence of a cointegration relationship between *ln_scale_drafter*, *sech*, and *patent*. Based on the ARDL model, we determined the optimal lag order of all three variables to be 1 via AIC and BIC. The regression results with *Δsech* as the dependent variable are shown in Table 7.

**Table 7:** Results of the PECM for scale efficiency.

Time interval		2007–2020	2007–2013	2014–2020
DV		*Δsech*	*Δsech*	*Δsech*
EC	*ln_scale_drafter*	0.0140***	0.0086	0.0203*
		(0.0054)	(0.0076)	(0.0114)
	*patent*	-0.0036***	0.0017	-0.0100***
		(0.0012)	(0.0018)	(0.0032)
ECM	*ec*	-0.9580***	-1.2947***	-0.4663***
		(0.0514)	(0.0710)	(0.0671)
	*Δln_scale_drafter*	-0.0036	-0.0038	-0.0082**
		(0.0028)	(0.0044)	(0.0038)
	*Δpatent*	0.0003	0.0078	-0.0048
		(0.0031)	(0.0052)	(0.0030)
	*Constant*	0.9294***	1.2235***	0.4702***
		(0.0607)	(0.0910)	(0.0817)
Method		*dfe*	*dfe*	*dfe*

Note: In cases where "initial replication failure" or circular iterations occurred during estimation, the dfe group average estimation method was used.

Throughout the length of time, *ln_scale_drafter* (*β =* 0.0140; *p<*0.01) had significant positive long-run effects on *Δpech* and *patent* had significant negative long-run effects. However, neither had significant short-run effects. This might indicate that standardization could boost scale efficiency in the long run and thus increase TFP. As there might be critical changes in economic, political, and financial aspects during 2007–2020, the relationship between the variables was likely to be structurally shifted accordingly. In this section, we divided the study period into two phases and ran each regression separately. The estimated coefficient of *ln_scale_drafter* (*β =* 0.0086; p > 0.1) was not significant for the period 2007–2013. This suggested that the drafters’ production scale did not exert long-term effects on regional scale efficiency during this time. In contrast, the results for 2014–2020 showed a significant positive long-run impact of *ln_scale_drafter* (*β =* 0.0203; *p<*0.1) on scale efficiency, while its short-run impact (*β =* -0.0082; *p<*0.01) was significantly negative.

This is also illustrated by the changing reliance on economies of scale in China’s economic development in recent years. The phrase "new normal" for China’s economy was formally introduced in May 2014. This indicated the Chinese government’s recognition that the country’s economic development should change from a size- and speed-oriented mode to a quality- and efficiency-oriented mode, i.e., to enhance production efficiency while maintaining the existing production scale. China’s economic development should strive for structural transformation that shifts the focus from factors to quality and efficiency. This is consistent with the TFP trend observed in this study. Around 2014, the contribution of standardization to scale efficiency showed a substantial difference, showing the economic system’s shift from merely extending the scale of production to enhancing the efficiency of current capacity.

Technological standards and economies of scale might mutually reinforce each other in a sufficiently stable technical and economic context. Due to the large market and the low labor cost, China rapidly developed to be one of the important industrial countries following WTO admission. However, the 2008 financial crisis and the ongoing trend of anti-globalization ultimately obstructed local and worldwide market growth, as well as international technological communication. The main obstacle to China’s economic reform might be a significant overcapacity problem. The situation would be exacerbated if we relied too heavily on large-scale manufacturing modes. As a result, industrial policies seem to orientation converge and industrial structure homogenize [96, 97]. From the standpoint of standards, standard innovation would be a feasible solution to this challenge. Developing countries, such as China, might target sophisticated foreign technologies to update domestic standards, encouraging local businesses to create, adopt, and implement these new standards in production and operation. A standard innovation strategy might lead to high-quality economic development and build new competitive advantages [98, 99].

## 6. Conclusions and implications

Following our theoretical study, we built a PECM utilizing China’s inter-provincial panel data from 2007 to 2020 to investigate the long and short-term relationships between standardization, R&D, and innovation-driven development. The following are the key findings: First, both standardization and R&D are critical engines of innovation-driven development. Such favorable driving impacts are long-lasting and consistent, although their functions vary by place. Second, standardization has the greatest impact on TFP through improving technical efficiency, whereas R&D drives both technical development and technical efficiency improvement. Third, while the influence of technical standard drafters’ production scale on scale efficiency was insignificant from 2007 to 2013, it became substantial after 2014 with China’s macroeconomic reform of "transforming the mode and changing the structure." This represented the Chinese government’s recognition that the country’s economic development should enhance production efficiency based on existing production scales. China’s economy is undergoing structural change, shifting from a factor-oriented to a quality-efficiency-oriented one.

To offer sustainable energy for economic growth in the context of the "new normal" of the economy, China would need to adopt an innovation-driven development plan. Standardization is becoming increasingly vital in enabling economic and commercial exchanges, encouraging scientific and technical advancement, and regulating social governance [100]. We concentrated on how standardization worked in TFP and its decomposition. This has important implications for developing countries engaged in implementing innovation-driven development. First, in innovation-driven development, standardization may be as vital as R&D. Enterprises have the right to focus on "technique patented + patent standardization" and actively lead or participate in the formulation and distribution of technical standards.

Social administrators should actively support economic agents’ standardization efforts, strengthen the building of standard data platforms, and encourage standard co-creation and co-sharing. Second, the important issue in the interaction between standardization and technical R&D is the alignment of their technological and time requirements. Economic entities should consider not just market acceptance, but also the interests of SEP licensees while developing standards [101–103]. Third, standardization actors in developing countries could greatly strengthen international standards cooperation, make their standards benchmark with the world’s best level, and utilize all their efforts to facilitate the "going abroad" of their standards to participate in international standard competitions [104, 105]. This might be a crucial strategy for improving the country’s economic competitiveness.
